# Non-Invasive Heart Failure Evaluation Using Machine Learning Algorithms

**DOI:** 10.3390/s24072248

**Published:** 2024-03-31

**Authors:** Odeh Adeyi Victor, Yifan Chen, Xiaorong Ding

**Affiliations:** School of Life Science and Technology, University of Electronic Science and Technology of China, Chengdu 610054, China; eddyvick59@gmail.com (O.A.V.); yifan.chen@uestc.edu.cn (Y.C.)

**Keywords:** heart failure, photoplethysmogram, echocardiogram, machine learning

## Abstract

Heart failure is a prevalent cardiovascular condition with significant health implications, necessitating effective diagnostic strategies for timely intervention. This study explores the potential of continuous monitoring of non-invasive signals, specifically integrating photoplethysmogram (PPG) and electrocardiogram (ECG), for enhancing early detection and diagnosis of heart failure. Leveraging a dataset from the MIMIC-III database, encompassing 682 heart failure patients and 954 controls, our approach focuses on continuous, non-invasive monitoring. Key features, including the QRS interval, RR interval, augmentation index, heart rate, systolic pressure, diastolic pressure, and peak-to-peak amplitude, were carefully selected for their clinical relevance and ability to capture cardiovascular dynamics. This feature selection not only highlighted important physiological indicators but also helped reduce computational complexity and the risk of overfitting in machine learning models. The use of these features in training machine learning algorithms led to a model with impressive accuracy (98%), sensitivity (97.60%), specificity (96.90%), and precision (97.20%). Our integrated approach, combining PPG and ECG signals, demonstrates superior performance compared to single-signal strategies, emphasizing its potential in early and precise heart failure diagnosis. The study also highlights the importance of continuous monitoring with wearable technology, suggesting a significant stride forward in non-invasive cardiovascular health assessment. The proposed approach holds promise for implementation in hardware systems to enable continuous monitoring, aiding in early detection and prevention of critical health conditions.

## 1. Introduction

Around 65 million people worldwide suffer from heart failure, a chronic, progressive, and incurable illness that causes about 7 million deaths annually [1]. Heart failure can manifest due to various factors, including inadequate myocardial relaxation, impaired ejection, or a combination of these issues. Furthermore, several underlying disorders such as coronary artery disease, hypertension, atrial fibrillation, heart valve irregularities, excessive alcohol consumption, infections, and idiopathic cardiomyopathy, in addition to structural heart abnormalities, can precipitate heart failure [2]. In individuals with heart failure, the heart loses its ability to effectively pump a sufficient volume of blood to meet the body’s organ and tissue oxygenation requirements [3].

The global incidence of heart failure is experiencing an upward trend, particularly in developed nations, constituting a significant public health concern [4]. In the United States, the present count of adults afflicted by heart failure stands at approximately 6.2 million, with a projected 46% increase anticipated by 2030 [5]. Factors contributing to this surge encompass an aging population, enhanced management of chronic illnesses, advancements in acute coronary syndrome treatments, and improved care for heart failure patients [6]. Europe is similarly affected, with an estimated 15 million individuals grappling with heart failure, leading to over 3 million hospitalizations annually. The substantial prevalence and recurrent hospitalization patterns associated with heart failure impose noteworthy economic burdens on both healthcare systems and society, with annual healthcare expenditures in the United States exceeding USD 30 billion [4].

Preventing heart failure and other cardiovascular diseases (CVDs) is significantly more successful when prevention and therapy are initiated promptly. Unfortunately, in the early stages of heart failure, many patients are asymptomatic, leading to missed opportunities for optimal treatment and an increased risk of complications. Nevertheless, certain physiological signals, such as electrocardiogram (ECG) and photoplethysmography (PPG), undergo alterations influenced by blood pressure levels [7,8]. These changes primarily manifest as morphological shifts in physiological signals, providing insights into the functional status of the heart and vascular system. Lifestyle modifications are integral to heart failure management, with dietary changes, exercise, and sodium restriction playing pivotal roles. Adherence to heart-healthy diets and engaging in regular physical activity contribute not only to symptom relief but also to increased life expectancy [9,10]. The INTERHEART study, exploring the impact of lifestyle factors on cardiovascular outcomes, highlights the profound influence of dietary habits and physical activity on overall cardiac health [11].

**Figure 1:**
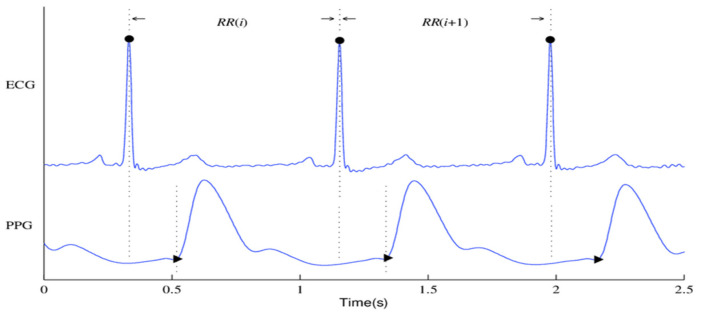
An ECG signal along with its corresponding PPG signal.

Figure 1 shows the ECG and PPG signals graphically. The waveforms of the ECG signal are shown at the top where the primary ECG peaks are indicated by dark circles. The PPG signal and associated waveform are shown beneath and the peaks corresponding to systole and diastole are shown by the dark circles in the subfigure.

Although so much research has been conducted on heart failure, most of them apply either echocardiogram or photoplethysmogram signals in their study. A paper introduced the concept of a non-invasive assessment method for the detection of ischemic heart disease patients from fingertip photoplethysmogram (PPG) signal. A unified feature set pertaining to heart rate variability (HRV) and PPG waveform morphologies was established to differentiate between individuals with and without CAD. For classification, they employed the support vector machine (SVM). Using a corpus of 112 people chosen from the MIMIC II dataset, their methodology achieves sensitivity and specificity ratings of 82% and 88%, respectively, in identifying CAD patients. They also obtained 73% and 87% sensitivity and specificity ratings from a different dataset of 30 patients who were gathered from an urban hospital utilizing a commercial oximeter device [12]. The findings from a study on a Hybrid Lightweight 1D CNN-LSTM architecture for automated ECG beat-wise classification offer promising implications for advancing the field of automated ECG classification, which makes it very suitable for embedded systems designs that can be used in clinical applications for monitoring heart diseases in a faster and more efficient manner [13]. A conditional Generative Adversarial Network (GAN) model (P2E-WGAN) was designed to reconstruct/synthesize realistic ECG signals from PPG signals; the results demonstrate the model’s potential for providing a paradigm shift in telemedicine by bringing ECG-based clinical diagnoses of cardiovascular disease to individuals via simple PPG assessment by wearables [14]. The synthesis of ECG waveforms from PPG signals using the P2E-WGAN approach has several potential applications and implications for clinical practice and medical device development such as enabling wearable devices equipped with PPG sensors to potentially provide continuous and long-term monitoring of ECG signals in daily life settings leading to the development of intelligent healthcare systems for clinical diagnoses of cardiac diseases and anomalies in real time through machine learning and cloud computing.

In another study, the authors introduced a non-invasive and cost-effective method for detecting coronary artery disease (CAD) via photoplethysmography (PPG), suitable for at-home monitoring. The analysis focuses on extracting distinguishing features from the time domain of the PPG signal and its second derivative. CAD patients were classified using a support vector machine-based classifier. The study evaluated the approach using ICU patient data from the MIMIC-II dataset and achieved a sensitivity of 85% and a specificity of 78% in identifying CAD patients [15]. A study was conducted on time-domain features from PPG signals to differentiate between subjects with and without diseases using various classification methods. The study evaluated ten metrics from the confusion matrix, and the Boosted Tree classifier outperformed the others, achieving an accuracy of 94%, sensitivity of 95%, specificity of 95%, and precision of 97% [16].

The UCI dataset repository was utilized to extract ECG features for use in a study that focused on heart failure prediction using the multi-criteria weighted vote-based classifier. The experimental outcomes validate the efficacy of the proposed ensemble classifier in handling a wide range of attribute types, with a notable high diagnostic accuracy of 87.37%. Furthermore, the classifier demonstrated impressive sensitivity at 93.75%, specificity at 92.86%, and an F-measure of 82.17%. These findings underscore the potential of this classifier as a valuable tool for accurate and comprehensive heart disease prediction [17]. A cross-domain joint dictionary learning (XDJDL) framework for synthesizing ECG waveforms from PPG signals was suggested in a study; the experimental results demonstrated the possibility of providing an affordable preliminary diagnosis screening from PPG signals and long-term, user-friendly ECG monitoring to help with early identification and screening for specific heart illnesses [18]. A Heart Disease Prediction System (HDPS) was developed aimed at assisting medical practitioners in diagnosing heart diseases. The system selects 13 relevant features from clinical data, constructs an artificial neural network based on these features, and creates a user-friendly interface. The HDPS offers output through various means, including ROC curve displays, execution time, accuracy, sensitivity, and specificity. Impressively, the HDPS achieved an 80% classification accuracy, indicating its potential as a valuable tool for heart disease diagnosis [19]. The detrended fluctuation analysis (DFA) method was used by Kamath et al. [20] to compute the short-term (20 s) ECG segments for CHF and normal hearts. The method produced 98.4% and 98% average sensitivity and specificity rates, respectively.

The combination of PPG and ECG signals for disease diagnosis has shown promising results. Yao et al. extracted 70 features from these signals for blood pressure estimation and used mutual information coefficient analysis to identify a highly discriminative feature subset [21]. Vandecasteele et al. utilized Heart Rate Variability (HRV) and Pulse Rate Variability (PRV) from ECG and PPG for predicting epileptic seizures, indicating the potential of these combined signals in seizure detection [22]. Additionally, ECG and PPG signals have been used to estimate respiratory sinus arrhythmia, revealing variations in heart rate due to breathing [23]. Findings from a study on the development and testing of a tri-modal device for monitoring cardiovascular parameters to aid in the diagnosis and monitoring of cardiovascular diseases emphasized the reliability and stability of devices in monitoring cardiovascular disease diagnosis [24]. In a study on alcohol consumption, Wang et al. combined ECG and PPG features, achieving a high accuracy of 95% in their study [25].

This study bridges a notable research gap by introducing a novel approach that integrates PPG and ECG signals for heart failure assessment. While previous studies have traditionally analyzed these signals independently, our innovative methodology leverages their combined power, offering a comprehensive evaluation of cardiac health. This approach not only enhances diagnostic accuracy but also holds the potential to detect heart failure at an earlier stage, promising to transform the field of cardiac healthcare. 

The integration of photoplethysmography (PPG) and electrocardiography (ECG) signals in our study serves to address the complementary nature of these modalities in assessing cardiovascular health [26]. ECG signals primarily focus on the electrical activity of the heart, providing detailed information about arrhythmias, electrical abnormalities, and cardiac rhythm [27]. However, ECG may lack direct insights into peripheral vascular resistance and the pulsatile component of blood flow. In contrast, PPG signals are sensitive to changes in peripheral vascular resistance and offer information about the pulsatile nature of blood flow [28]. Integrating multiple signals enhances the reliability of the monitoring system. In case one sensor faces limitations or artifacts, the other can provide complementary information, reducing the risk of false negatives or positives. Wearable technologies incorporating both PPG and ECG sensors enable efficient arrhythmia detection, remote monitoring, and early detection of heart failure, potentially reducing morbidity and mortality rates associated with cardiovascular diseases [27,29]. By combining both signals, our assessment model aims to compensate for the limitations of each modality. The integration allows us to comprehensively evaluate both electrical and hemodynamic features, providing a more accurate assessment and understanding of heart function and contributing to the accuracy of heart failure assessment. The rationale behind using both signals lies in their synergistic ability to capture a broader spectrum of physiological features, ensuring a more robust evaluation compared to relying on either signal independently. For instance, in the case of heart failure, ECG might indicate arrhythmias, whereas PPG could unveil signs of impaired cardiac output. By coalescing these insights, the clinician gains a more comprehensive understanding of the heart’s performance and potential issues. Combining the attributes of both ECG and PPG signals is imperative to harness the comprehensive advantages derived from their respective features.

The contribution of this paper can be summarized in the following three points:

- This paper introduces an innovative approach by integrating photoplethysmography (PPG) and electrocardiogram (ECG) signals for heart failure assessment. This integration leverages the unique strengths of both non-invasive monitoring methods to enhance diagnostic accuracy and enable early detection of heart failure.
- The study underscores the clinical relevance of this integrated approach, emphasizing its potential to improve patient care, offer personalized treatment plans, and reduce healthcare costs. By preventing advanced heart failure complications, it has the potential to generate substantial cost savings for healthcare systems.
- The significant improvements achieved by the proposed integrated method in contrast to the results obtained from individual ECG and PPG signals underscore the potency of combining these two modalities. This not only enhances diagnostic accuracy but also highlights the potential for early detection in the assessment and management of heart failure.

The rest of this paper follows this structure: The study’s methodology, which includes data collecting, signal processing, feature extraction, feature importance analysis and selection, and classification, is covered in detail in Section 2. Section 3 is dedicated to presenting the outcomes and discussions of various classification models and distinct feature sets. Finally, Section 4 provides an in-depth exploration of the strengths and limitations of this work, while Section 5 offers a comprehensive conclusion that highlights the clinical relevance of our study’s findings.

## 2. Materials and Methods

**Figure 2:**
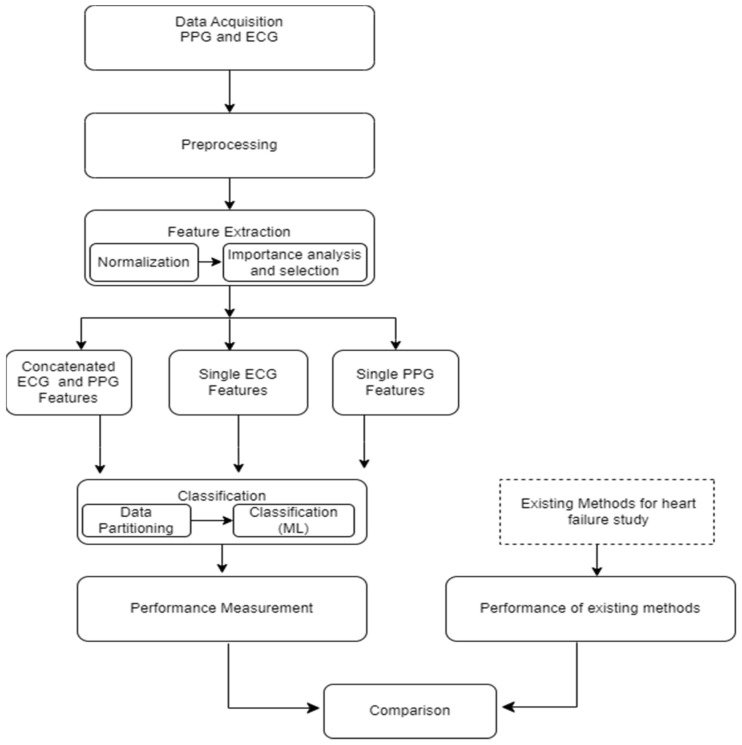
General framework/architecture of the proposed study.

The foundational block diagram of our proposed approach for heart failure assessment is shown in Figure 2, which involves the integration of PPG and ECG signals and employs machine learning algorithms for classification. The methodology encompasses the following steps: (i) acquisition of ECG and PPG signals as the primary inputs of the algorithm; (ii) preprocessing of the ECG and PPG signals which include denoising and eliminating artifacts; (iii) extraction of informative features from the preprocessed signals; (iv) normalization of the dimension of the extracted features; (v) feature importance analysis and selection; (vi) partitioning and classification of the data; and, ultimately, (vii) comparative evaluation with prior research and studies. The subsequent subsections go into further depth about each of these blocks.

### 2.1. Dataset and Signal Pre-Processing

The data utilized in this research were sourced from the MIMIC-III (Medical Information Mart for Intensive Care) database, a comprehensive repository containing information from a large cohort of Intensive Care Unit (ICU) patients [30]. All the physiological data utilized in this study were obtained simultaneously for a coherent representation of cardiovascular dynamics from the Philips CareVue Clinical Information System, with data collected from models M2331A and M1215A. We obtained proper consent for data extraction from MIMIC-III for research purposes, as indicated by Record ID 51903504, and adhered to ethical guidelines by completing the web-based training course provided by the National Institutes of Health Protecting Human Research Participants. The dataset comprises a total of 1636 instances, including 954 control subjects and 682 heart failure patients with varying degrees of heart failure, ranging from mild to severe. The patients were identified based on ICD-9 codes related to heart failure, ensuring a broad spectrum of cardiac pathologies. This approach aimed to enhance the robustness and sensitivity of our presented method to different stages of heart failure. By incorporating a diverse range of cases, our machine learning model was trained to recognize patterns and features across the continuum of cardiac damage, enabling a more comprehensive evaluation. The inclusion of patients with varying levels of heart failure is a deliberate choice to enhance the model’s sensitivity to different stages of heart failure. As shown in Table 1 (below), the inclusion criteria involved patients aged 20 years or older at the time of ICU admission. Exclusions were made for patients below this age, those lacking an ICU record, or missing data for echocardiography or photoplethysmogram. The mean age of the included patients was 51.6 ± 9.9 years, with 41.1% being women and 58.9% men. 

Three main sources of interference generally endanger the quality of the ECG signal: (A) the power-line noise at 50 or 60 Hz, which makes up the majority of the noise power in these signals and is essentially stationary; (B) the signal’s baseline wandering, which is referred to as the breathing artifact and manifests as a low-frequency component in the time domain (this can cause analog circuitry to become saturated or lose some of its effective precision, which can erode the accuracy of digitalization); (C) non-stationary, high-frequency noises resulting from muscle contractions. We created a preprocessing block to filter and denoise the signals in order to eliminate the degrading impacts of noise and artifacts from the raw signals. First, we addressed baseline wander and motion artifacts by applying a high-pass filter with a cut-off frequency of 150 Hz. This filter effectively removed low-frequency components related to baseline drift and motion artifacts while retaining the higher-frequency components essential for heart function and cardiac event analysis. Notably, recordings with abnormalities or noise, such as missing peaks, pulsus bisferiens, no signal (sensor-off), etc., were excluded. The retained signal fragments had more than 30,000 points, equivalent to 4 min of data at a 125 Hz adoption rate.

### 2.2. Feature Extraction

Feature extraction constitutes the procedure of uncovering meaningful patterns and insights within raw data, thereby crafting a more informative representation that refines the accuracy of prognosis and diagnosis [31]. In the realm of machine learning and data analysis, feature extraction revolves around the conversion of input data into a collection of features suitable for utilization as inputs in models or algorithms. The features extracted from physiological signals play a crucial role in the accuracy and specificity of disease estimation or diagnosis. Various methods, such as the wavelet approach [32], have been employed for feature extraction. In specific cases, deep learning approaches, like the artificial neural network—long short-term memory (ANN-LSTM) network, have been utilized for extracting features from successive ECG and PPG signals, demonstrating efficacy in estimating blood pressure [33]. Other studies have employed an extensive feature extraction approach combining PAT features with PPG features for hypertension prediction [34]. Additionally, calibration methodologies have been applied in some studies for cuffless blood pressure estimation, effectively eliminating correlations between subjects’ blood pressure and pulse transit time (PTT), particularly suitable for short intervals and applications like monitoring blood pressure during exercise tests [35]. These diverse approaches highlight the importance of tailored feature extraction methods based on the specific diagnostic requirements or health parameters under consideration. In the present study, MATLAB software (version R2022b), designed and distributed by MathWorks (Natick, MA, USA), was employed for conducting the feature extraction process.

The feature extractor block is responsible for extracting three distinct categories of informative features from both PPG and ECG signals. The features extracted are broadly classified into three groups: The first category is centered on physiological parameters, including metrics such as augmentation index, heart rate, arterial stiffness index, and heart rate variability parameters (pNN50, NN50, RMSSD, and SDNN); physiological features delve into parameters related to the body’s physiological responses. The second group of features encompasses amplitude-related attributes, such as pulse pressure, systolic pressure, diastolic pressure, and P-wave characteristics; these features provide insights into the signal’s strength and intensity. The third category involves interval-related features, including peak-to-peak interval, QRS interval, and RR interval, the interval features offer information about the durations between specific points within the signal.

**Figure 3:**
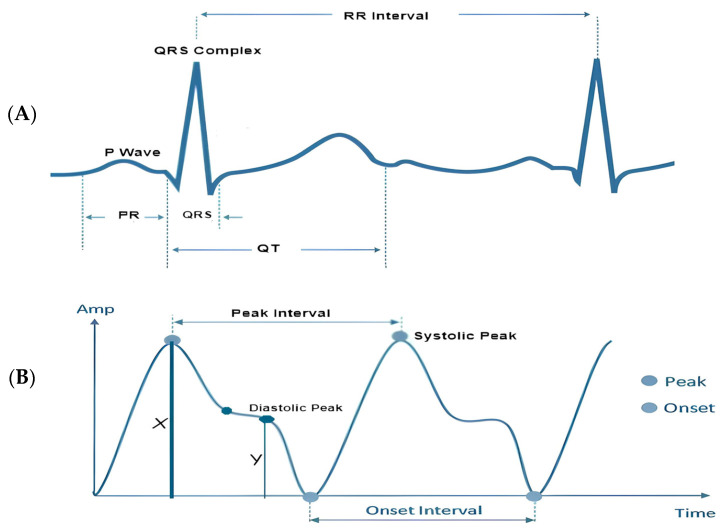
Visual representation of the extracted features: (**A**) visual representation of features extracted from a single ECG signal; (**B**) visual representation of features extracted from a single PPG signal.

In accordance with the physiological underpinnings of heart failure and its relationship with ECG and PPG signals, we identified and extracted 13 pivotal features. These features encapsulate essential cardiovascular information and were extracted from both ECG and PPG signals within each cardiac cycle to facilitate heart failure evaluation. The extracted characteristics are represented visually in Figure 3, while Table 2 furnishes a comprehensive overview of each feature’s class and the clinical importance/implications. It is essential to highlight that the PPG features extracted in this study are solely based on the identification of three readily discernible points, specifically the peak of the first derivative of PPG, the foot, and the peak of the PPG waveform.

Table 3 (below) demonstrates how morphological changes in cardiovascular features serve as indicators of structural or functional irregularities in the heart. In individuals with chronic heart failure (CHF) and acute myocardial infarction (AMI), heart rate variability in the time domain provides valuable prognostic information. Key parameters include the standard deviation of normal beat intervals (SDNN) and pNN50, representing the percentage of adjacent NN intervals differing by more than 50 ms. An SDNN value of less than 50 ms or pNN50 lower than 3% is indicative of high risk, 50 to 100 suggests moderate risk, and a value over 100 ms or a pNN50 greater than 3% is considered normal [36]. The QRS interval, another predictor of heart failure, generally ranges between 0.06 and 0.12 ms in healthy individuals. A prolonged QRS interval may indicate delays in the ventricular depolarization process. The RR interval, denoting the time between consecutive R waves in the QRS signal, is a critical parameter for assessing ventricular rate. In healthy individuals, normal ECG values for the RR interval typically range between 0.6 and 1.2 s. Prolonged RR intervals, defined as > 1.5 s, are commonly observed in patients with atrial fibrillation [37]. 

Pulse pressure, representing the difference between systolic and diastolic blood pressure, and systolic pressure, an indicator of pulsatile changes in blood volume due to arterial blood flow, typically range between 0.5 and 10 mmHg and 80 and 120 mmHg, respectively, in healthy subjects. During heart failure, the arterial system undergoes changes, leading to increased stiffness. Elevated augmentation index values are indicative of increased wave reflections, reduced arterial compliance, and impaired vascular function, all of which are associated with heart failure. Structural and electrical changes in the heart can affect atrial function, which in turn causes P-wave abnormalities, such as increased duration or altered shape, which may signify atrial remodeling, a common feature in heart failure patients.

Heart rate (HR) also serves as a predictor of cardiovascular, cerebrovascular, and all-cause mortality [38]. A normal resting heart rate for adults ranges between 60 and 100 beats per minute. Increased heart rate has been associated with elevated cardiovascular risk and total mortality. The relationship between increased heart rate and adverse cardiovascular events remains significant even after adjusting for major cardiovascular risk factors, indicating the independent prognostic value of heart rate in various populations and clinical conditions.

### 2.3. Feature Normalization

To address the scale differences in features extracted from PPG and ECG signals, which represent distinct heart failure indicators, this study utilized min–max normalization on the entire dataset. This crucial preprocessing step ensured that all feature values were uniformly scaled within a range of 0 to 1, preventing analytical inaccuracies and anomalies during model training. The normalization method employed followed a straightforward mapping equation to achieve this standardization:

(1) $$\mathrm{Xnorm}\;=\frac{x-\min\left(x\right)}{\max\left(x\right)-\min\left(x\right)}$$

This process not only promotes model stability and efficiency but also mitigates the impact of outliers, enhancing the reliability of our heart failure evaluation model. 

### 2.4. Feature Importance Analysis and Feature Selection

**Figure 4:**
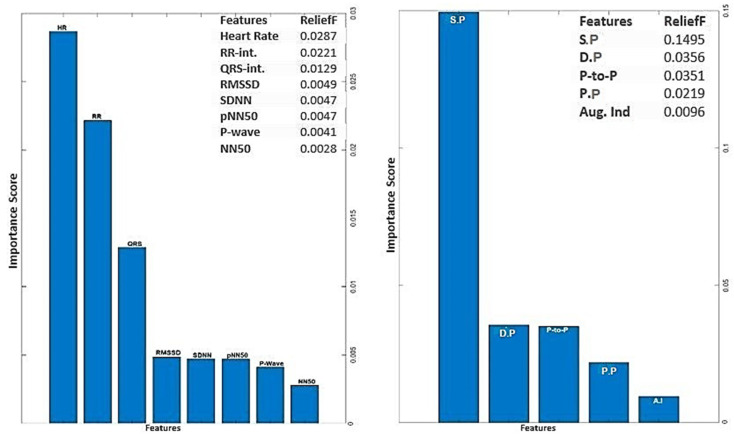
Feature importance score for ECG and PPG signals using ReliefF algorithm.

**Figure 5:**
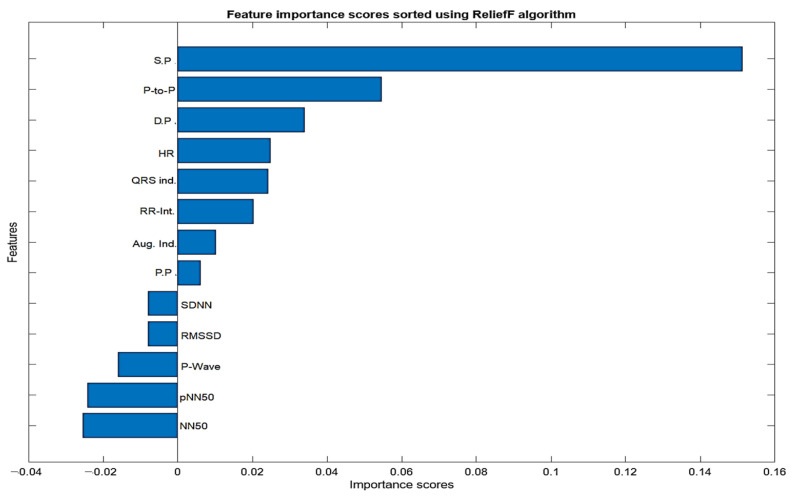
Importance score for combined features from PPG and ECG signals.

The input feature vectors for both PPG and ECG were further reduced using the relief feature algorithm (ReliefF) [39,40]. The ReliefF algorithm is a filter-style feature selection technique that estimates weights by taking the nearest neighbor into account. In practical applications, this enhanced relief derivative, referred to as ReliefF, is the most frequently utilized version [41]. The study employed the ReliefF algorithm to further reduce input feature vectors derived from both PPG and ECG signals. ReliefF is a well-established feature selection method known for its robust performance in multi-class classification scenarios and its capacity to handle noisy datasets with missing values. This improved variant, ReliefF, incorporates k nearest neighbors (KNN) from each class to estimate feature weights, enhancing the accuracy of weight estimation, particularly in noisy dataset settings [42]. The study initially used the ReliefF algorithm to assess the relevance of features within PPG and ECG signals individually as illustrated in Figure 4 (below). This analysis identified key features within each modality for heart failure assessment, aiding in the determination of which modality (ECG or PPG) offered better discriminatory power. However, this individual analysis had a limitation in that it might not capture interactions or synergies between PPG and ECG features. To overcome this limitation, the study conducted a combined feature importance analysis on a feature set that included both ECG and PPG data (illustrated in Figure 5 below). This holistic approach provided a comprehensive view of feature importance, taking into account the contributions of both modalities. This comprehensive perspective offered insights into the significance of individual features within each signal and the collective impact of combining features from both PPG and ECG. Ultimately, this holistic view sheds light on the roles of each modality and highlights the potential advantages of integrating them in the context of heart failure evaluation.

The analysis of ECG data illuminates the pivotal role played by several key features as discriminators among different classes, particularly in individuals with heart failure. The heartbeat feature, reflecting the frequency of heartbeats, serves as a fundamental indicator of cardiac activity, with deviations signaling disruptions in the pumping function. The RR interval, indicative of the time between successive R-peaks, offers insights into heart rate variability, highlighting irregularities in cardiac rhythm. The QRS interval, representing ventricular depolarization duration, provides information on the heart’s electrical conduction system. Features such as RMSSD, SDNN, and pNN50, which gauge short-term variability, overall variability, and the percentage of significant variations, respectively, offer crucial information on autonomic function and cardiovascular regulatory mechanisms. Altered patterns in these features among individuals with heart failure contribute to a comprehensive understanding of the physiological changes associated with the condition. The examination of feature importance derived from PPG signals accentuates the clinical relevance of systolic pressure, diastolic pressure, peak-to-peak interval, and pulse pressure in the PPG waveform for heart failure assessments. These features bear intricate connections to the heart function. In the realm of heart failure, where cardiac function is frequently compromised, alterations in the morphology of these features serve as indicative markers of the physiological changes associated with the condition. Systolic pressure, representing the maximum arterial pressure during systole, and diastolic pressure, indicating the minimum arterial pressure during diastole, offer a comprehensive view of blood pressure dynamics. The peak-to-peak interval captures variations between consecutive peaks, reflecting the pulsatile nature of blood flow. Pulse pressure, as a fundamental feature in the PPG waveform, provides information about the strength and regularity of the pulsatile signal. Comprehending the subtle variations in these characteristics greatly enhances the clinical applicability of PPG-based assessments by contributing to the nuanced assessment of heart failure. The selected features above for PPG (four features) and ECG (six features) were used for classifications involving the analysis of independent signals.

The feature selection process for the integration of PPG and ECG signals involved a meticulous examination of absolute values to identify features of substantial significance in the context of heart failure assessment. From the initial pool of extracted features, a refined set of ten (10) features was selected. Notably, systolic pressure, diastolic pressure, peak-to-peak interval, NN50, pNN50, P-wave, heart rate, QRS complex, RR interval, pulse pressure, and augmentation index emerged as pivotal contributors to the classification task. The consideration of absolute values was paramount, ensuring a comprehensive evaluation of these features and capturing the essential dynamics of the cardiovascular system. These selected features exhibit notable clinical relevance, aligning with established physiological indicators of heart failure. ReliefF played a key role in highlighting their importance, emphasizing their ability to distinguish patterns associated with heart failure. The chosen features contribute to the overall effectiveness and clinical relevance of the heart failure assessment model, enhancing its interpretability and accuracy in classifying instances of the condition.

### 2.5. Data Partitioning and Classical Machine Learning

Data partitioning is a crucial step in supervised machine learning, aiding in the training, optimization, and validation of predictive models. Various techniques exist for partitioning datasets into subsets, each suited to different dataset sizes. In this study, the datasets (n = 1636) were split into 75% (1227) training sets and 25% (409) testing sets. The intentional design of the control group with a larger sample size aimed to provide a more balanced representation of the real-world distribution, enhancing the reliability and accuracy of our analysis. To ensure the model’s stability and generalization, a 10-fold cross-validation (CV) procedure was applied to the training data before model optimization. During this process, the training data were divided into ten equal-sized ‘folds.’ The model was trained and validated ten times, with each fold taking turns as the validation set while the others were used for training. By evaluating the model’s performance across different training data subsets, this method helped prevent overfitting. Also, extensive hyper-parameter tuning to optimize the model’s performance while guarding against overfitting and under-fitting was carried out. The utilization of the cross-validation strategy mentioned earlier and ensemble methods further contributed to the robustness of our model. Our commitment to avoiding over-parametrization was manifested in the comprehensive evaluation of various training aspects, emphasizing a balanced trade-off between model complexity and generalization. This meticulous approach allows for a fair and reliable comparison between the integrative and single-input models. The performance of the resulting model was evaluated on a different test set, giving an assessment of its generalization to completely unknown data.

In this study, nine (9) machine learning algorithms were implemented for classification and performance analysis using the Weka software (Version 3.9.6). Weka (Waikato Environment for Knowledge Analysis) is a cross-platform open source, renowned for its popularity in the realm of machine learning. Developed by the University of Waikato, this Java-based software (Java 11.0) offers a versatile platform for various data analysis and machine learning tasks [43].

Each machine learning algorithms were implemented in this study based on its specific strengths and suitability for the study’s goals, including handling complex features, dealing with noisy data, and providing insights into feature importance or relationships within the dataset; the combination of these diverse algorithms also allows for a comprehensive evaluation of heart failure using the extracted PPG and ECG features. 

## 3. Results and Discussion

As previously described, the evaluation of each model involves a 10-fold cross-validation (CV) of dataset samples, ensuring that there is no overlap between the training and testing data. To assess the classification performance, precision, recall, accuracy, and F-measure were computed using the following metrics:

(2) $$\mathrm{Accuracy}\;=\frac{\mathrm{TP}+\mathrm{TN}}{\mathrm{TP}+\mathrm{TN}+\mathrm{FP}+\mathrm{FN}}$$

(3) $$\mathrm{Specificity}=\frac{\mathrm{TN}}{\mathrm{TN}+\mathrm{FP}}$$

(4) $$\mathrm{Sensitivity}\;\left(\mathrm{Recall}\right)=\frac{\mathrm{TP}}{\mathrm{TP}+\mathrm{FN}}$$

(5) $$F1\mathrm{-score}=\frac{2\;\times \;(\mathrm{Recall}\;\times \;\mathrm{Precision})\;}{\mathrm{Recall}+\mathrm{Precision}}$$

In this context, TP denotes a set of correctly identified test results, FP represents a set of test results incorrectly identified, TN signifies a set of correctly rejected test results, and FN stands for a set of test results incorrectly rejected. For this study, we computed and compared the results from a single ECG signal and a single PPG signal with the results obtained from the integration of both PPG and ECG signals for heart failure evaluation.

### 3.1. Result from Classification with Features Extracted from Single PPG Signal

The features extracted from the PPG signals were employed to compute the performance metrics using the various machine learning algorithms discussed in the previous section. PPG signals inherently capture dynamic alterations in blood volume and vascular attributes, thus providing distinctive insights into cardiovascular health from an alternative vantage point. The outcomes of this approach were highly promising, as evident below.

**Figure 6:**
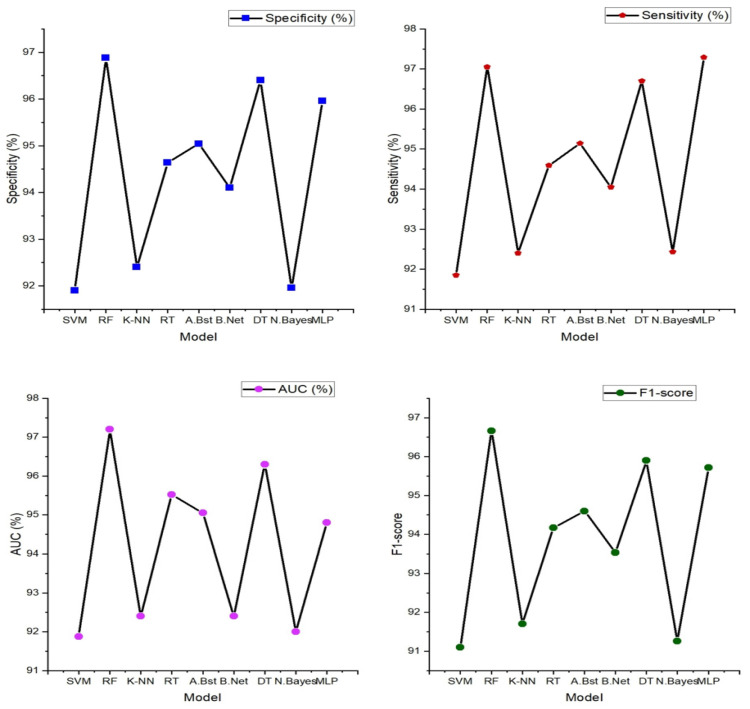

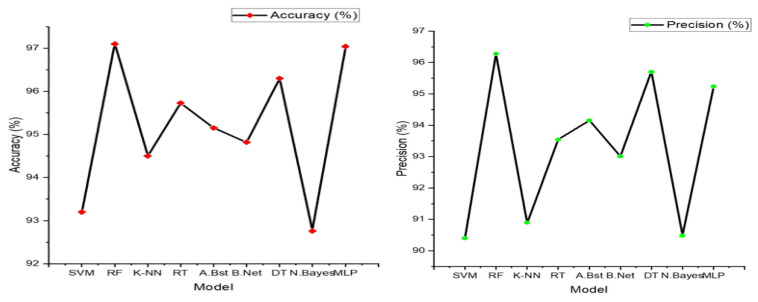
Heart failure (HF) classification performance (specificity, sensitivity, accuracy, precision, AUC, and F1-score) with features extracted from a single PPG signal.

From Figure 6 below, Random Forest stands out as the top performer with an impressive accuracy of 97.10%, sensitivity of 97.05%, specificity of 96.88%, precision of 96.28%, AUC value of 97.20%, and F1-score of 96.66%, indicating its strong ability to correctly classify individuals with and without heart failure. It also excels in sensitivity, specificity, precision, and AUC, demonstrating its comprehensive effectiveness in evaluating heart failure. While Random Forest takes the lead, it is worth noting that other models, such as support vector machine (SVM), K-nearest neighbor, and decision tree, also deliver commendable performances with accuracies ranging from 93% to 96%. However, Random Forest consistently outperforms these alternatives in most performance metrics, reinforcing its position as the optimal choice for heart failure classification.

### 3.2. Result from Classification with Features Extracted from Single ECG Signal

The result presented in the figure below shows the various machine learning models’ performance obtained from the classification of features extracted from a single ECG signal. 

**Figure 7:**
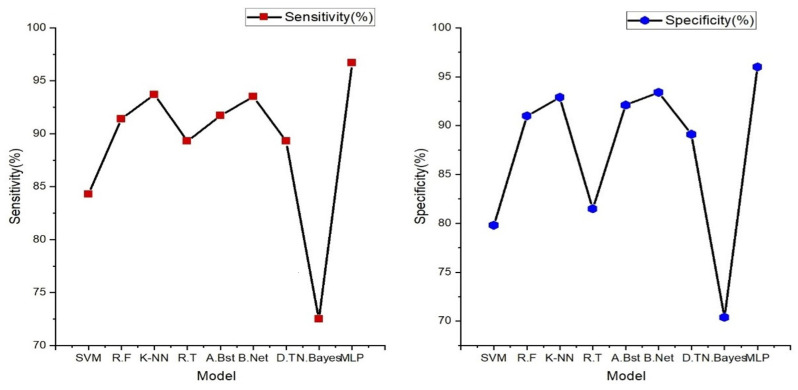

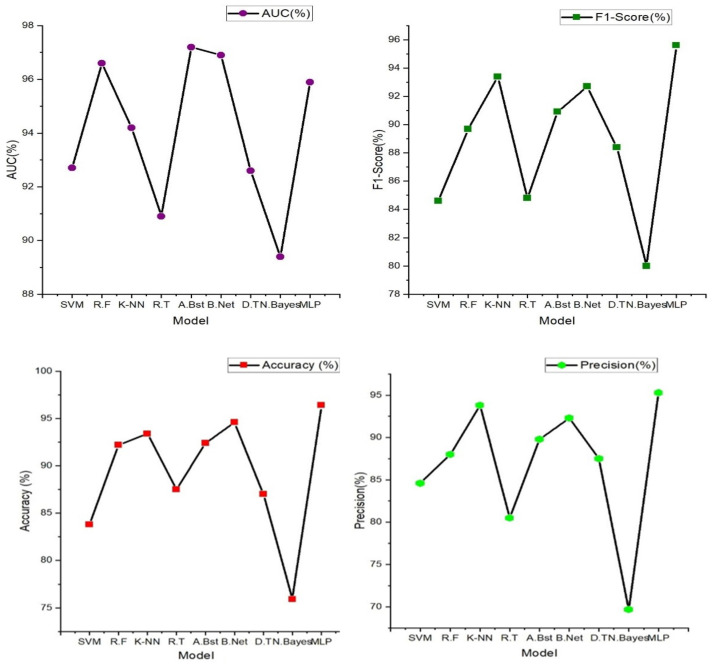
Classification performance (specificity, sensitivity, accuracy, precision, AUC, and F1-score) with features extracted from a single ECG signal.

In Figure 7 (below), the findings prominently highlight the exceptional performance of the multi-layer perceptron (MLP), a feedforward artificial neural network characterized by its multiple layers of interconnected nodes. It showcases the highest accuracy at 96.40%, underscoring its impressive capability in accurately discerning individuals with and without heart failure. Furthermore, the MLP model excels across various evaluation metrics, including sensitivity (96.70%), specificity (96.00%), precision (95.30%), and F1-score (95.60%). These results signify its comprehensive effectiveness in classifying heart failure cases with precision. Notably, other models such as K-nearest neighbor, AdaBoost, and Random Tree also deliver commendable performances, achieving accuracies in the range of 84% to 92%. These models exhibit a balanced trade-off between sensitivity and specificity, demonstrating their ability to identify heart failure cases while keeping false positives at a minimum.

### 3.3. Result from Classification of Integrated Features Extracted from PPG and ECG Signals

Integrating ECG and PPG signals offers a significant advantage, as it harnesses the wealth of complementary information they provide. ECG, being a gold standard for assessing heart rate and rhythm irregularities, is adept at capturing electrical activity. On the other hand, PPG captures changes in blood volume and vascular characteristics, offering insights into cardiovascular health from a different perspective. By combining these signals, we amalgamate intricate details of the circulatory system, resulting in a more holistic evaluation that transcends the limitations of individual signals. The result obtained from the classification of the novel approach of combining these signals is represented below.

Table 4 (below) shows the comparison of these evaluation/performance metrics on the selected machine learning models. From the displayed result, the support vector machine model outperforms other machine learning models. The evaluation/performance metrics; accuracy, sensitivity, specificity, precision, AUC, and F1-score obtained are 98%, 97.60%, 96.90%, 97.20%, 98.40%, and 97.70%. Figure 6 displays the radar plot of these performance metrics achieved in the various machine learning algorithms.

### 3.4. Comparison of Results Obtained

Performance metrics obtained from this novel approach to the integration of the PPG and ECG signal were also tested and compared to the results obtained from the performance of PPG and ECG signals independently. Figure 6 and Figure 7 illustrate the performance results obtained from the classification performed on these signals independently. 

In comparison with the performance result obtained from the integration of these features, it is evident that the integration of these signals for heart failure study provides unique insights into cardiovascular health from an alternative vantage point.

The table above demonstrates the comparison between the results obtained from the analysis of the integration of PPG and ECG signals versus the results obtained from the ECG and PPG signals in isolation. When PPG data were considered in isolation, the Random Forest model emerged as the top performer, achieving an accuracy of 97.10%. This model demonstrated remarkable sensitivity, specificity, precision, and an F1-score, all hovering around the 96–97% range. These metrics collectively indicated its strong potential for accurate heart failure classification.

In contrast, the ECG data were effectively evaluated using the MLP (Multilayer Perceptron) model, yielding an accuracy of 96.40%. While its accuracy was slightly lower than that of PPG, MLP exhibited a well-balanced trade-off between sensitivity and specificity, making it a valuable contender in heart failure assessment.

It can also be noted that the result obtained from using the PPG signal outperformed the result from using only the ECG signals; this can be attributed to some factors such as PPG signals being less susceptible to interference from electrical sources and electronic equipment compared to ECG. This characteristic ensures that PPG measurements remain reliable and consistent in various environments, which is particularly beneficial in settings where electrical interference may be present, allowing for dependable monitoring and accurate assessment of heart failure without external disruptions. Also, PPG excels in evaluating peripheral hemodynamics, offering valuable insights into blood circulation beyond the heart, and it effectively measures changes in blood volume in peripheral blood vessels, shedding light on the efficiency of circulation and peripheral perfusion. Given that HF often affects peripheral blood flow, this capability is pivotal for understanding the broader cardiovascular dynamics associated with the condition. Also, in terms of motion tolerance, PPG’s resilience to motion artifacts is a notable advantage, particularly for individuals with heart failure. Patients with HF may experience limited mobility or discomfort, and PPG’s ability to maintain measurement accuracy even during subtle movements ensures that vital data can be reliably collected. This motion tolerance enables continuous monitoring without undue disruption, a crucial aspect in assessing HF patients’ condition.

However, the most notable findings arose from the integration of both PPG and ECG signals, where the Random Forest model demonstrated exceptional performance. This integrated approach resulted across various evaluation metrics, including accuracy (98.00%) sensitivity (97.60%), specificity (96.90%), precision (97.20%), AUC (97.70%), and F1-score (98.40%), suggesting that the combination of these signals significantly enhanced the model’s capability for heart failure detection. Moreover, an outstanding AUC of 97.70%, indicates a superior ability to discern heart failure cases while minimizing false positives.

These outcomes highlight the potential of integrating PPG and ECG data for more accurate heart failure assessment. These results reveal superior performance compared to the use of these signals independently. Particularly, when assessed using the Random Forest model, the integrated approach exhibited exceptional accuracy and overall effectiveness, highlighting its potential significance in heart failure evaluation.

### 3.5. Comparison with Other Works

The performance of the proposed approach was also assessed in comparison to prior studies that independently employed ECG and PPG for various applications. Our findings indicate that the proposed method exhibited superior performance when contrasted with these studies, which separately utilized ECG and PPG modalities for their distinct analyses. The results of this comparative analysis are detailed in Table 5 below.

From the results below (Table 6), in comparison to previous ECG-focused studies, the current study achieved high-performance metrics ranging between 96.40%, 96.70%, 96.00%, 95.130%, and 95.90%. Notably, the multi-layer perceptron (MLP) model in the current study outperformed the majority of previous ECG-based studies in terms of accuracy, sensitivity, specificity, and precision.

For PPG analysis, the current study achieved high accuracy, specificity, and sensitivity, with values ranging from 96.88% to 97.10%. These results outperform several previous PPG-focused studies in terms of accuracy, sensitivity, and specificity. The Random Forest (RF) model stood out with the highest accuracy.

The integration of PPG and ECG signals in the current study demonstrated promising results, with accuracy ranging from 91.20% to 98.00%. These results surpassed the majority of both ECG and PPG-focused studies, indicating that the combination of these signals provides a substantial benefit in heart failure assessment. The Random Forest (RF) model achieved the highest accuracy in this integrated approach, further emphasizing its effectiveness in comprehensive heart failure evaluation.

## 4. Clinical Application Prospect 

This study presents substantial clinical relevance. By combining the unique strengths of these non-invasive monitoring methods, healthcare providers can significantly enhance the accuracy and timeliness of heart failure diagnosis. The results obtained from our study demonstrate the significant potential benefits of integrating PPG and ECG signals for heart failure evaluation. With an impressive accuracy of 98%, sensitivity of 97.60%, specificity of 96.90%, and precision of 97.20%, the integrated approach outperforms the results obtained from individual ECG and PPG signals. These findings suggest that the integrated approach holds promise for early, precise, and non-invasive diagnosis of heart failure. The high sensitivity implies effective identification of individuals with heart failure, contributing to early intervention and improved patient outcomes. This non-invasive evaluation method not only enhances patient care through timely diagnosis but also has the potential to reduce healthcare costs by enabling more targeted interventions. Furthermore, the accurate classification of heart failure cases paves the way for personalized treatment strategies, tailoring medical interventions based on individual patient needs. Overall, our study provides compelling evidence supporting the potential benefits of integrating PPG and ECG signals for heart failure assessment, aligning with the envisioned advantages mentioned in the abstract.

Moreover, the non-invasive nature of PPG and ECG signals allows for telemedicine and remote monitoring, enhancing patient accessibility and addressing healthcare challenges in remote areas. It also has the potential to reduce healthcare costs by preventing advanced heart failure complications. Additionally, this integrated approach fosters ongoing research and development in cardiac healthcare, promising advanced diagnostic and monitoring tools. In summary, the integration of PPG and ECG signals has the potential to revolutionize heart failure diagnosis and management, offering early detection, personalized care, cost savings, and improved patient outcomes.

## 5. Limitations, Future Work and Conclusions

Despite its merits, the integration of PPG and ECG signals faces several limitations. The quality and availability of PPG and ECG data can vary, impacting the reliability of the approach. Technical expertise is required for implementing and interpreting this integrated approach, which may not be readily available in all healthcare settings. Access to the necessary equipment for data collection can be limited in certain healthcare facilities, particularly in resource-constrained settings. Additionally, PPG and ECG signals are sensitive to motion artifacts and environmental interference, which can affect data quality. Advancements in wearable sensor technologies have made significant strides in mitigating these challenges. Modern wearable devices are increasingly equipped with advanced algorithms and hardware designed to reduce noise and compensate for motion-related artifacts. This makes them increasingly viable for collecting reliable ECG and PPG data even as patients engage in their daily activities. 

It is also important to note the exploration of deep learning methods stands as a potential avenue for future research, balancing advancements with the unique demands of interpretability in medical applications. With the development of deep learning and other algorithms that need high computing power, its learning capacity to automatically learn intricate features from the data could be advantageous, which not only enables the analysis process to no longer require a feature extraction using hand-crafted techniques but it also has great advantages in accuracy and robustness, particularly is sufficient training data are provided. However, we emphasize the importance of interpretability in the medical domain and the challenges associated with acquiring extensive labeled datasets.

Our research primarily analyzed signals recorded in a resting state, as is typical in an intensive care unit (ICU) setting, which is the environment from which the MIMIC III data was obtained. We recognize that this approach may not capture certain cardiac anomalies that manifest specifically during physical exertion. However, we believe that the high accuracy of our method even with resting state data holds promising implications for future applications, including integration with wearable technology. With wearables, it is feasible to monitor ECG and PPG signals during various activities, potentially allowing for the detection of exercise-induced cardiac events. We anticipate that our approach, when applied to such dynamic data, could yield equally promising results.

Handling of patient data raises concerns about data privacy and security, and future works should implement stringent data protection protocols, such as employing advanced encryption techniques for data storage and transmission and strictly adhering to international standards such as HIPAA (Health Insurance Portability and Accountability Act) and GDPR (General Data Protection Regulation). Additionally, access controls will be put in place to ensure that only authorized personnel can access patient data.

## Figures and Tables

**Table 1:** Subject characteristics (n = 1636).

	Heart Failure (682)	Non-Heart Failure Patients (954)
Age [yr]		
20–40	29.7 ± 5.7	24.08 ± 4.7
40–60	50.5 ± 5.9	56.66 ± 9.5
>60	74.6 ± 8.8	54.60 ± 10.1
Gender		
Male	402 (58.9%)	444 (46.6%)
Female	280 (41.1%)	509 (53.4%)
Weight		
Male	78.8 ± 17.6	78.5 ± 17.5
Female	78.6 ± 17.4	78.4 ± 17.9
Height [cm]	172.3 ± 13.7	169.4 ± 11.9
BMI [Kg/m^2^]	26.5 ± 9.7	27.5 ± 3.2

**Table 2:** Summary of the features and the clinical importance/implications.

Class of Feature	Features	Information Provided
Class 1 (Amplitude features)	Pulse Pressure Systolic Pressure Diastolic Pressure P-wave	Monitoring these amplitude features over time can provide insights into the progression of heart failure and the effectiveness of therapeutic interventions aimed at managing vascular resistance.
Class 2 (Interval information)	Peak-to-peak interval QRS interval RR interval	Changes in these intervals can indicate alterations in cardiac function and hemodynamics associated with heart failure. Researchers can gain insights into the pathophysiology of heart failure and assess the severity of the condition.
Class 3 (Physiological features)	Augmentation Index HRV Parameters (pNN50, NN50, RMSSD, and SDNN) Heart Rate	They offer insights into heart function, blood flow, arterial stiffness, and autonomic nervous system activity.

**Table 3:** Features and the normal values for a healthy adult.

Feature	Description	Duration	Disease Diagnosis
Pulse pressure	Difference between the systolic and diastolic blood pressure	0.5–10 mmHg	Atherosclerosis Congestive heart failure
Systolic pressure	Indicator of the pulsatile changes in blood volume caused by arterial blood flow	80–120 mmHg	Artery stiffness Heart failure
P-wave	Atrial depolarization	0.08–0.11 s	Heart failure
Diastolic pressure	Represents the amplitude of the signal during the diastolic phase of the cardiac cycle	<80 mm	Ischemic heart disease Cardiomyopathy
Peak-to-peak interval	Represents the duration between successive peaks in a signal	0.6–1.2 s	Atrial fibrillation Heart failure
RR interval	The interval between two successive R-waves of the QRS complex ventricular rate	0.6–1.2 s	Paroxysmal atrial fibrillation Congestive heart failure
Augmentation index	The difference between systolic and diastolic blood pressure	20–80	Heart failure
Heart rate	A measure of the number of times the heart contracts or beats within a specific time frame, usually one minute	60–100 bpm	Heart failure Atrial fibrillation
QRS interval	Ventricular depolarization	0.08–0.11 s	Heart failure Tachycardia Acute coronary syndrome
RMSSD NN50 pNN50	Shows how active the parasympathetic system is relative to the sympathetic nervous system	19–48 ms 5–25 ms 5–18%	Heart failure Hypertension Arrhythmia Coronary artery disease

**Table 4:** Performance of various machine learning models for heart failure (HF) classification with the integration of ECG and PPG signals.

Model	Performance Metrics					
	Accuracy (%)	Sensitivity (%)	Specificity (%)	Precision (%)	AUC (%)	F1-Score (%)
SVM	98.00	97.60	96.90	97.20	98.80	97.70
Random Forest	96.80	96.70	96.90	96.20	99.60	96.40
K-NN	94.90	79.30	95.70	94.80	95.30	86.20
Random Tree	96.90	96.70	96.80	96.20	96.80	98.50
AdaBoost	96.90	96.80	96.80	85.10	99.60	91.87
BayesNet	95.50	95.70	95.50	94.60	96.80	95.20
Decision Tree	96.00	95.70	96.40	95.70	96.80	95.70
NaiveBayes	91.20	91.30	91.00	89.40	95.20	90.30
MLP	96.50	96.80	96.50	95.70	99.80	96.30

**Table 5:** Comparison between the best performing models from isolated signals vs. performance from the integration of both signals.

	Model	Performance Metrics					
		Accuracy (%)	Sensitivity (%)	Specificity (%)	Precision (%)	F1Score (%)	AUC (%)
PPG	Random Forest	97.10	97.05	96.88	96.28	97.20	96.66
ECG	MLP	96.40	96.70	96.00	95.30	95.90	95.60
Integration	SVM	98.00	97.60	96.90	97.20	98.40	97.70

**Table 6:** Comparison of results obtained from the existing literature.

Author	Dataset	Signal	Features Extracted	Algorithm	Acc. (%)	Sens. (%)	Spec. (%)	Pre. (%)	F1-Score (%)
Simge et al. [44]	UCI 300	ECG	Chol, trestbps, fbs, restecg, slope	Cubic SVM Linear SVM DT Ensemble	52.3 67.3 67.7 67.0	-	-	-	-
Ali et al. [45]	UCI	ECG	RestECG, Trestbps, Chol, fbs	KNN SVM NaïveBayes	80 83 84	-	75 77 80	80 82 83	-
Shouman et al. [46]	CHDD	ECG	Chol, trestbps, fbs, restecg	GRDT NaïveBayes KNN	79.1 83.5 83.2	75.6 78.0 76.7	81.6 80.8 85.1	- - -	- - -
Tu et al. [47]	UCI	ECG	Chol, trestbps, fbs, restecg	Bagging DT	81.41 78.91	74.93 72.01	86.64 84.48	- -	- -
Bashir et al. [17]	CHDD 303	ECG	Chol, trestbps, fbs, restecg	Ensemble NaïveBayes DT SVM	81.82 78.79 76.57 86.67	73.68 68.49 63.58 73.68	92.86 92.86 71.24 79.51	- - - -	82.17 73.61 71.51 65.10
Pal et al. [16]	50	PPG	Crest-time, augmentation index, pulse pressure, S.P/D.P	BT SVM KNN LR	94 85 83 83	95 83 79 83	95 87 82 85	97 83 97 82	96 87 89 85
Banerjee et al. [12]	MIMIC II 112	PPG	Systolic peak, NN interval, HRV	SVM	-	82	88	-	-
Paradhker et al. [15]	MIMIC II 55	PPG	Augmentation index, stiffness index	SVM	-	85	78	-	-
Current Study	MIMIC III 1636	ECG	QRS interval, RR interval, HRV, heart rate	MLP	96.40	96.70	96.00	95.30	95.90
		PPG	S.P, D.P, P.P, P-to-P	RF	97.10	97.05	96.88	91.20	96.66
		Integration of PPG and ECG signal		SVM	98.00	97.60	96.90	97.20	97.70

Note: Acc.: accuracy, Spec.: specificity, Sens.: sensitivity, Pre.: precision, KNN: K-nearest neighbor, BT: Boosted Tree, SVM: support vector machine, MLP: multi-layer perceptron, LR: logistic regression, DT: decision tree, RF: Random Forest. GRDT: Gain ratio decision tree, CHDD: Cleveland Heart Disease Dataset. S.P: systolic pressure, D.P: diastolic pressure, P-to-P: peak-to-peak amplitude, and P.P: pulse pressure.

## Data Availability

The authors do not have permission to share data.
